# Characterization of Three Pleiotropic Drug Resistance Transporter Genes and Their Participation in the Azole Resistance of *Mucor circinelloides*

**DOI:** 10.3389/fcimb.2021.660347

**Published:** 2021-04-14

**Authors:** Gábor Nagy, Sándor Kiss, Rakesh Varghese, Kitti Bauer, Csilla Szebenyi, Sándor Kocsubé, Mónika Homa, László Bodai, Nóra Zsindely, Gábor Nagy, Csaba Vágvölgyi, Tamás Papp

**Affiliations:** ^1^ Department of Microbiology, Faculty of Science and Informatics, University of Szeged, Szeged, Hungary; ^2^ MTA-SZTE “Lendület” Fungal Pathogenicity Mechanisms Research Group, Department of Microbiology, University of Szeged, Szeged, Hungary; ^3^ Department of Biochemistry and Molecular Biology, Faculty of Science and Informatics, University of Szeged, Szeged, Hungary

**Keywords:** *Mucor*, pleiotropic drug resistance, CRISPR-Cas9, azole, ABC transporter

## Abstract

Mucormycosis is a life-threatening opportunistic infection caused by certain members of the fungal order Mucorales. This infection is associated with high mortality rate, which can reach nearly 100% depending on the underlying condition of the patient. Treatment of mucormycosis is challenging because these fungi are intrinsically resistant to most of the routinely used antifungal agents, such as most of the azoles. One possible mechanism of azole resistance is the drug efflux catalyzed by members of the ATP binding cassette (ABC) transporter superfamily. The pleiotropic drug resistance (PDR) transporter subfamily of ABC transporters is the most closely associated to drug resistance. The genome of *Mucor circinelloides* encodes eight putative PDR-type transporters. In this study, transcription of the eight *pdr* genes has been analyzed after azole treatment. Only the *pdr1* showed increased transcript level in response to all tested azoles. Deletion of this gene caused increased susceptibility to posaconazole, ravuconazole and isavuconazole and altered growth ability of the mutant. In the *pdr1* deletion mutant, transcript level of *pdr2* and *pdr6* significantly increased. Deletion of *pdr2* and *pdr6* was also done to create single and double knock out mutants for the three genes. After deletion of *pdr2* and *pdr6*, growth ability of the mutant strains decreased, while deletion of *pdr2* resulted in increased sensitivity against posaconazole, ravuconazole and isavuconazole. Our result suggests that the regulation of the eight *pdr* genes is interconnected and *pdr1* and *pdr2* participates in the resistance of the fungus to posaconazole, ravuconazole and isavuconazole.

## Introduction

Mucorales species are ubiquitous saprophytes (Hoffmann et al., 2013), but several species are also known as opportunistic human pathogens, which can cause a life-threatening infection commonly called as mucormycosis. The most common mucormycosis-causing agent is *Rhizopus delemar* (also referred to as *R. arrhizus* var. *delemar* and *R. oryzae*) (Walther et al., 2019), which can cause mucormycosis worldwide, followed by *Lichtheimia*, *Apophysomyces*, *Rhizomucor*, *Mucor* and *Cunninghamella* species (Prakash and Chakrabarti, 2019). Besides the pathogenesis of *R. delemar*, those of *Lichtheimia corymbifera* and *Mucor circinelloides*, have also been studied (Riley et al., 2016; Skiada et al., 2018; Jeong et al., 2019). Clinical manifestations of mucormycosis include rhino-cerebral, pulmonary, cutaneous, and disseminated forms in immunocompromised patients (Skiada et al., 2018; Jeong et al., 2019). These infections are generally associated with rapid progression and high mortality rates (i.e. from 20 to 100% depending on the underlying condition, the clinical presentation, and the treatment), furthermore, their diagnosis and treatment are often difficult (Riley et al., 2016; Skiada et al., 2018). The immunocompromised state due to malignancy (Ibrahim et al., 2012; Riley et al., 2016; Skiada et al., 2018) or immunosuppressive treatments (Bakr et al., 2008; Ibrahim et al., 2012), diabetic ketoacidosis (Roden et al., 2005; Rammaert et al., 2012; Roilides et al., 2012), corticosteroid treatment (Prabhu and Patel, 2004), malnutrition (Sun and Singh, 2011), burn and trauma (Riley et al., 2016) are the main risk factors for the infection. For the treatment of mucormycosis, international guidelines recommend systemic antifungal therapy in combination with surgical intervention (Caramalho et al., 2017; Skiada et al., 2018). The first-choice antifungal agent against Mucorales fungi is amphotericin B. Besides, application of two triazoles, posaconazole and isavuconazole are approved for second-line or salvage therapy (Riley et al., 2016). However, the spectrum of applicable drugs is extremely narrow as these fungi are intrinsically resistant to most of the routinely used antifungal agents including most azoles (Almyroudis et al., 2007; Drogari-Apiranthitou et al., 2012; Riley et al., 2016).

The specific target of azoles is a cytochrome P450 enzyme, the lanosterol 14α-demethylase enzyme (Cyp51 or Erg11), which catalyzes an intermediate step of the ergosterol biosynthesis, the conversion of lanosterol to 4,4-dimethyl-cholesta-8,14,24-trienol. Ergosterol is the main sterol component of the fungal cell membrane. Blocking of its synthesis causes accumulation of toxic 14α-methyl sterols, which alter the stability and permeability of the cell membrane (Odds et al., 2003). Development of antifungal resistance is a complex process and depends on either host- and microbial related factors (White et al., 1998). In the case of azoles, the resistance mechanisms most frequently include alterations in ergosterol biosynthetic enzymes and up-regulation of multidrug transporters (Cowen et al., 2015).

Expression of drug efflux pumps, which belong to the ATP-binding cassette (ABC) or the major facilitator superfamily (MFS) transporters, was found to a major factor contributing in the fungal resistance to azoles in several human pathogenic fungi (Prasad and Rawal, 2014; Holmes et al., 2016; Lopez-Ribot et al., 2017; Sharma and Chowdhary, 2017). In *Candida albicans*, two ABC transporters belonging to the pleiotropic drug resistance (PDR) protein class of ABC transporter superfamily, *Candida* drug resistance 1 (Cdr1) and Cdr2 were found to have the primary role in the azole resistance (Rocha et al., 2017). *Cdr* genes responsible for azole efflux have also been identified and characterized in other *Candida* species (Rocha et al., 2017). Overexpression of the *Cryptococcus neoformans* antifungal resistance 1 (AFR1) protein, which also belongs to the PDR family, led to resistance to fluconazole and an increased virulence in mouse models (Chang et al., 2018). In the case of *Aspergillus fumigatus*, the role of the ABC transporter Cdr1B in itraconazole, voriconazole and posaconazole resistance was proven (Fraczek et al., 2013; Hagiwara et al., 2017), but other ABC transporter genes were also found to be upregulated after azole treatment (Lopez-Ribot et al., 2017).

Little is known about the background of azole resistance in mucormycosis-causing fungi. It is assumed that specific amino acid substitutions in one of the Cyp51 enzymes can affect the sensitivity of these fungi to several azoles, such as fluconazole or voriconazole (Caramalho et al., 2017). However, the role of drug efflux pumps in azole resistance have not been examined in this fungal group. *M. circinelloides* belongs to the order Mucorales and it is a widely used model organism in genetic and molecular biological studies, among others on light sensing (Corrochano and Garre, 2010; Vellanki et al., 2018; Yu and Fischer, 2019; Navarro et al., 2020), molecular regulation and signal processes (Lee et al., 2015; Ruiz-Vázquez et al., 2015; Calo et al., 2017; Lax et al., 2020), morphogenesis (Lee et al., 2015; Moriwaki-Takano et al., 2020; Vellanki et al., 2020), carotenogenesis (Zhang et al., 2016; Alcalde et al., 2019) and pathogenicity (Li et al., 2011; López-Fernández et al., 2018; Patiño-Medina et al., 2019). In this study, transcription of the eight genes of *M. circinelloides* possibly encoding PDR-type ABC transporters was analyzed and three of them (named as *pdr1*, *pdr2* and *pdr6*) were knocked out by CRISPR-Cas9 mutagenesis. Using the knock-out mutants, the effect of these transporters on the azole resistance was examined.

## Materials and Methods

### Strains, Media, and Growth Conditions

*Mucor circinelloides* strain MS12 (*leuA^-^* and *pyrG^-^*) (Benito et al., 1992) was used in the study. For nucleic acid extraction from *Mucor*, 10^6^ sporangiospores were plated onto solid minimal medium (YNB; 10 g glucose, 0.5 g yeast nitrogen base without amino acids (Sigma-Aldrich), 1.5 g (NH_4_)_2_SO_4_, 1.5 g sodium glutamate and 20 g agar per liter) supplemented with leucine and/or uracil (0.5 mg/ml) if required. In some cases, RNA extraction was performed after cultivation in 30 ml RPMI-1640 without agar (Biosera); the inoculum size was 10^4^ sporangiospores/ml. Fungal cultures were grown for 4 days under continuous light at 25°C.

To test the effect of azoles on the gene expression, after cultivating the fungus in 10 ml liquid RPMI-1640 for 24 h at 25°C, it was treated with the corresponding azoles and incubated for another 16 h before the RNA extraction. Ketoconazole (Alfa Aesar), itraconazole (Across Organics), fluconazole (Alfa Aesar), ravuconazole (Sigma Aldrich) and isavuconazole (Sigma Aldrich) were applied in a final concentration of 8 μg/ml, while the final concentration of posaconazole (Sigma Aldrich) was 2 μg/ml. Anaerobic growth was performed in a BBL GasPak Anaerobic System (Becton Dickinson) at 25°C. For the antifungal susceptibility test, *Candida krusei* ATCC 6258 was used as a reference strain. After cultivating *Mucor* in liquid RPMI-1640 for 24 h at 25°C, it was treated with the corresponding azole (i.e., ketoconazole, itraconazole, fluconazole, posaconazole, ravuconazole or isavuconazole), and incubated for another 16 h before RNA extraction.

To measure the colony diameters of the strains, 10^4^ sporangiospores were inoculated onto the center of solid YNB plates. The diameter of the colonies was measured daily after incubating the plates at 25°C using the MS12 strain as the growth control. In the experiment, we measured the colony diameter of two colonies using three biological and two technical replicates.

### Molecular Techniques

General procedures for plasmid DNA preparation, cloning and transformation of *E. coli* DH5α were performed by following standard methods (Sambrook et al., 1989). Plasmid DNA was isolated using the PureYield Plasmid Miniprep System (Promega).

Genomic DNA and RNA samples were purified from mycelia using the ZR Fungal/Bacterial DNA MiniPrepTM (Zymo Research) and the Quick-RNA MiniPrep kit (Zymo Research), respectively, according to the manufacturers’ instructions. Genes were amplified by PCR using the Phusion Flash High-Fidelity PCR Master Mix (Thermo Scientific) and the primers presented in **Supplementary Table 1**. Primer and oligonucleotide sequences were designed using the *M. circinelloides* CBS277.49v2.0 genome database (DoE Joint Genome Institute; http://genome.jgi-psf.org/Mucci2/Mucci2.home.html) (Corrochano et al., 2016).

### Sequence Analysis

Sequencing was commercially performed by LGC Genomics (Berlin, Germany). Amino acid sequences obtained from *M. circinelloides* CBS277.49v2.0 genome database and *Cryptococcus neoformans* Afr1 (UniProtKB: Q8X0Z3) were aligned using the ClustalW program (Higgins et al., 1996). BLAST searches were performed at the site of the National Center for Biotechnology Information (NCBI) (https://blast.ncbi.nlm.nih.gov/Blast.cgi.

Programs used to analyze the amino acid sequences of the putative PDR proteins were accessed throught the Swiss Expasy Server (http://www.expasy.ch) (Gasteiger et al., 2005). Domain search and prediction were performed using the Motif Scan (MyHits) program (Pagni et al., 2007).

Homologous sequences of *Cryptococcus neoformans* var. *neoformans* JEC21 AFR1 were searched on the JGI MycoCosm portal (https://mycocosm.jgi.doe.gov/mycocosm/home) (Grigoriev et al., 2014). Sequences with at least 50% identity were retrieved and hits from non-public genomes were removed. The remaining sequences were clustered by using MMseqs2 v. bbd564172bd55d9e6acd1170e59790c37157a21b (Steinegger and Söding, 2017). The sensitivity of the cascaded clustering was set to 7.5 applying the cluster reassign mode. Maximum Likelihood analysis was conducted with the cluster comprising of 853 sequences including the AFR1 protein and the 8 examined PDR proteins from *M. circinelloides* CBS 277.49 (for the sequences included see **Supplementary Table 2**). Sequences were aligned using MAFFT v. 7.453 (Katoh and Standley, 2013) with the E-INS-i iterative refinement method. Poorly aligned regions were removed by using trimAl v. 1.2rev57 (Capella-Gutierrez et al., 2009) with the -automated1 option. Phylogenetic reconstruction was carried out by using IQ-TREE v. 1.6.12 (Nguyen et al., 2015) with the LG4M+R6 model determined by the inbuilt model selection tool (Kalyaanamoorthy et al., 2017). Branch supports were calculated with 5000 ultrafast bootstrap (Hoang et al., 2018).

### Real-Time Quantitative Reverse Transcription PCR Analysis

Reverse transcription was carried out with the Maxima H Minus First Strand cDNA Synthesis Kit (Thermo Scientific) using random hexamer and oligo (dT)18 primers, following the instructions of the manufacturer. The qRT-PCR experiments were performed in a CFX96 real-time PCR detection system (Bio-Rad) using the Maxima SYBR Green qPCR Master Mix (Thermo Scientific) and the primers presented in **Supplementary Table 1**. The relative quantification of the copy number and the gene expression was achieved with the 2^-ΔΔCt^ method (Livak and Schmittgen, 2001) using the actin gene of *M. circinelloides* as a reference (Nagy et al., 2014). Experiments were performed in biological and technical triplicates.

### Design of the gRNAs and Construction of the Disruption Cassettes to Disrupt the *pdr* Genes by the CRISPR-Cas9 Method

The protospacer sequences designed to target the DNA cleavage in *pdr1* (CBS277.49v2.0 genome database Protein Id: 48059), *pdr2* (CBS277.49v2.0 genome database Protein Id: 83305) and *pdr6* (CBS277.49v2.0 genome database Protein Id: 146716) genes were the followings, 5′- tttatgaacttgtgtatgaa - 3′, 5′- tgctgatttcgagcgtatcac -3′ and 5’- ctatattgctcaagtcatca -3’, respectively. Using these sequences, the Alt-R CRISPR crRNA and Alt-RCRISPR-Cas9 tracrRNA molecules were designed and purchased from Integrated DNA Technologies (IDT). To form the crRNA:tracrRNA duplexes (i.e. the gRNAs), the Nuclease-Free Duplex Buffer (IDT) was used according to the instructions of the manufacturer. Genome editing strategies followed the set-up described earlier (Nagy et al., 2017). Homology direct repair (HDR) was applied for all gene disruptions following the strategy described previously (Nagy et al., 2017). Disruption cassettes functioning also as the template DNA for the HDR were constructed by PCR using the Phusion Flash High-Fidelity PCRMaster Mix (Thermo Scientific). At first, two fragments, upstream from start codon and downstream from stop codons of the targeted gene and the *M. circinelloides pyrG* gene (CBS277.49v2.0 genome database Protein Id: 36136) or *M. circinelloides leuA* gene (CBS277.49v2.0 genome database Protein Id: 33992) along with its own promoter and terminator sequences were amplified using gene specific primer pairs (see **Supplementary Table 1**). The amplified fragments were fused in a subsequent PCR using nested primers (see **Supplementary Table 1**); the ratio of the fragments in the reaction was 1:1:1.

### Construction of the pPdr1compI Plasmid Used to Complement the Deletion of the *pdr1* Gene

The *leuA* gene (CBS277.49v2.0 genome database ID: Mucci.e_gw1.2.132.1) using gene specific primers was amplified from the genomic DNA of the MS12 strain (**Supplementary Table 1**) and ligated into pJET1.2/blunt plasmid (Thermo Scientific) to create the plasmid pJet+leuA. The *pdr1* gene was amplified together with its own promoter and terminator regions using gene specific primers (**Supplementary Table 1**) and the resulting fragment was also ligated into pJet1.2/blunt vector to create the plasmid pJet+pdr1. Then, *pdr1* together with its regulatory regions was cut from this plasmid by the enzyme *Not*I and ligated at the same restriction site of pJet+leuA to construct pPdr1compl plasmid (**Supplementary Figure 1A**). This plasmid was used to introduce to the MS12- Δ*pdr1* to create MS12-Δ*pdr1*+pPdr1compl strains. The complemented *pdr1* gene was amplified by PCR using Mc48059P7 and Mc48059P8 primers (**Supplementary Figure 1B** and **Supplementary Table 1**).

### Transformation Experiments

For the CRISPR-Cas9-mediated gene knock out and the complementation of the Δ*pdr1* gene, the PEG-mediated protoplast transformation method was used according to van Heeswijck and Roncero (1984). Protoplasts were prepared as described earlier (Nagy et al., 1994). For the gene disruption by CRISPR-Cas9 method, 5 μg template DNA (disruption cassette), 10 μM gRNA and 10 μM Cas9 nuclease were added to the protoplasts in one transformation reaction as described by Nagy et al. (2019). To complement the deletion of the *pdr1*, 5 μg pPdr1compl plasmid DNA was added into one transformation reaction. In each case, transformants were selected on solid YNB medium by the complementation of the uracil and/or leucine auxotrophy of the MS12 strain. From each primary transformant, monosporangial colonies were formed under selective conditions. Deletion of genes was confirmed by sequencing in all cases while the absence of the transcripts of the deleted genes was proven by qRT-PCR.

### Measurement of Energy-Dependent Rhodamine 6G Efflux

Measurement of R6G efflux was analyzed according to Gbelska et al. (2017). Sporangiospores of the strains (10^7^) were inoculated in 10 ml YNB and incubated in a fridge for 16 h. The spores were then incubated for 4 h at 25°C under continuous shaking at 220 rpm. Germinating spores were collected by centrifuging the samples for 10 min at 4°C at 2000×*g*. The pellets were washed two times with ice-cold PBS buffer and 2-deoxy-D-glucose and R6G were added to the cells in final concentrations of 2 mM and 100 µM, respectively. Fungal cells were incubated for 2 h at 25°C in dark, washed two times with ice cold PBS and finally resuspended in PBS. Then, D-glucose were added to the samples, in a final concentration of 2 nM. After incubation for 10, 20 and 30 min, 400 µl of the samples were centrifuged for 2 min at 8000×*g* and 100 µl of the supernatants were transferred into a 96-wells black plate. The fluorescence signal was measured using a fluorimeter (FLUOstar OPTIMA, BMG Labtech) where the excitation filter was 520BP1 and the emission filter was 590-10. Experiments were performed in biological and technical triplicates. To determine the R6G concentration, a standard curve was generated using the following concentrations: 0, 100, 250, 500 and 1000 nM.

### Susceptibility Tests

Sensitivity of the fungal strains to different antifungal agents, and H_2_O_2_ was examined in a 96-well microtiter plate assay. The susceptibility test was performed according to the CLSI recommendation (Rex and Clinical, 2008) in three biological replicates. Ketoconazole (Alfa Aesar), itraconazole (Across Organics), fluconazole (Alfa Aesar), ravuconazole (Sigma Aldrich), posaconazole (Sigma Aldrich), isavuconazole (Sigma Aldrich), amphotericin B (Sigma Aldrich), hygromycin B (Sigma Aldrich), terbinafine (Sigma Aldrich), micafungin (Sigma Aldrich) and cyclohexymide (PanReac AppliChem) were dissolved in DMSO to prepare the stock. These stocks were then diluted with liquid RPMI-1640 medium. Final concentrations of ketoconazole, itraconazole, fluconazole, terbinafine and micafungin in the wells ranged from 0.125 to 64 μg/ml while those of ravuconazole and isavuconazole ranged from 0.125 to 16 μg/ml. In the case of posaconazole and amphotericin B, the range was 0.125 to 8 μg/ml. Cyclohexymide were diluted in RPMI-1640 to obtain a range of 250-0,78 μg/ml. Hydrogen peroxide was diluted in liquid RPMI-1640 to prepare a stock solution of 100 mM. Final concentrations of the hydrogen peroxide in the wells ranged from 0 to10 mM. Inocula were prepared and diluted in liquid RPMI-1640. Plates were incubated for 48 h at 25°C.

### Statistical Analysis

All measurements were performed in at least two technical and three biological replicates. Significance was calculated with paired t-test or One-way Anova using the Graph Pad Prism 7 program. P values less than 0.05 were considered as statistically significant.

## Results

### Characteristic Features of PDR Proteins of *Mucor circinelloides*

In the *M. circinelloides* genome database (DoE Joint Genome Institute; *M. circinelloides* CBS277.49v2.0; http://genome.jgi-psf.org/Mucci2/Mucci2.home.html), eight potential PDR proteins were found by amino acid similarity search using the amino acid sequence of *Cryptococcus neoformans* Afr1 (UniProtKB: Q8X0Z3) and *Candida albicans* Cdr1p (UniProtKB: P43071). The identity of amino acid sequences was between 36.8 and 39.6%. Despite the low identity values, the characteristic motifs of the 8 putative Pdr proteins could be identified. Protein ID, location and amino acid length are shown in **Table 1**. In all eight putative PDR proteins, the amino acid motifs characteristic to the PDR family, such as the first and second Walker-A and B sequences, the first and second ABC signature motifs (Posteraro et al., 2003) and the conserved PDR specific NBD1 (T/SL/FLK/RT/V/II/L) and NBD2 (TLLN/DC/VL/R) motifs (Rawal et al., 2016) were identified (**Supplementary Figure 2**). PDR/CDR ABC motifs (Gauthier et al., 2003) were also identified in all eight putative PDR proteins using the motif scan (MyHits) program (**Table 1**).

**Table 1:** The eight identified pleiotropic drug resistance protein genes and their location in the genome of *M. circinelloides*.

Protein ID	Name in this study	Location	Amino acid length	Position of PDR/CDR ABC motif	Position of NBD1	Position of NBD2
48059	PDR1	scaffold_03:2555182-2559793 (-)	1416 aa	667-805 aa	146-151 aa	831-837 aa
83305	PDR2	scaffold_06:2898818-2903484 (+)	1481 aa	737-872 aa	217-222 aa	898-904 aa
141912	PDR3	scaffold_04:1521204-1525513 (-)	1415 aa	669-807 aa	155-160 aa	833-839 aa
142239	PDR4	scaffold_04:2922648-2927179 (-)	1441 aa	698-833 aa	183-188 aa	859-865 aa
145852	PDR5	scaffold_06:2231882-2236192 (+)	1371 aa	628-763aa	108-113 aa	789-795 aa
146716	PDR6	scaffold_06:2892916-2897373 (+)	1384 aa	636-774 aa	115-120 aa	800-806 aa
158611	PDR7	scaffold_01:3472584-3477196 (-)	1463 aa	725-863 aa	193-198 aa	878-884 aa
186086	PDR8	scaffold_01:475770-480189 (-)	1349 aa	601-739 aa	80-85 aa	765-771 aa

Comparing the amino acid sequences,PDR6 and PDR7 proved to be the most similar proteins followed by PDR2 and PDR5 showing an amino acid identity of 82.79 and 82.49%, respectively.

**Figure 1:**
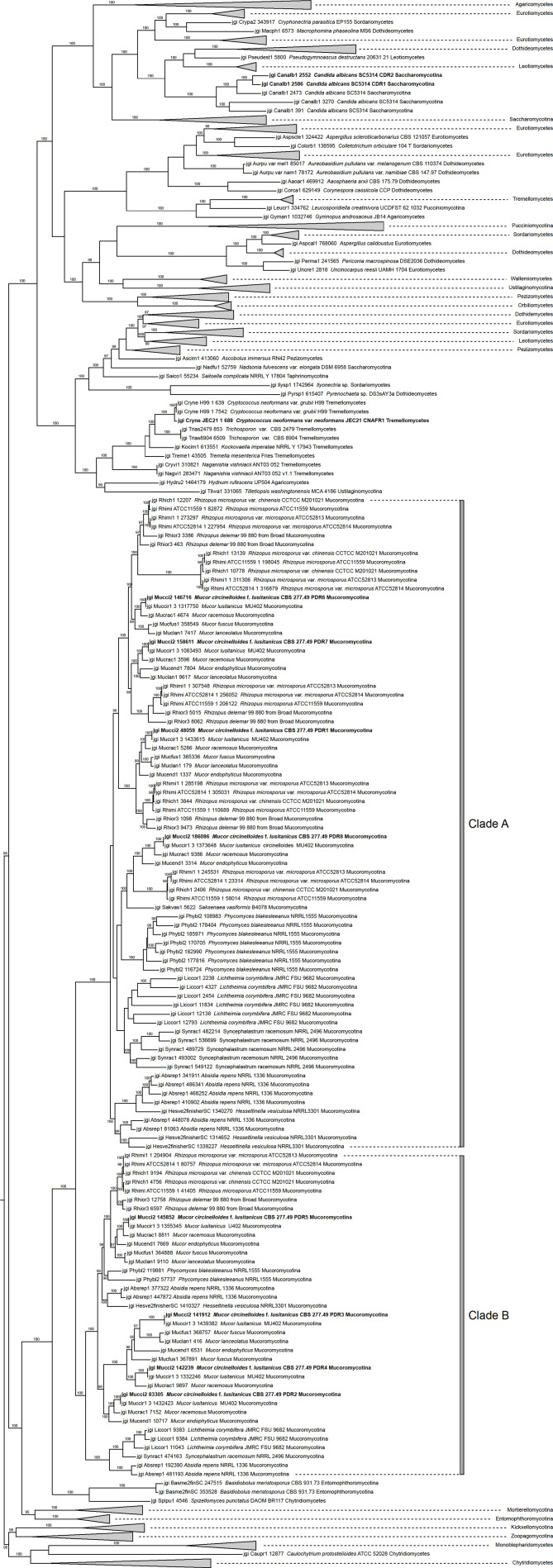
Phylogenetic tree of PDR proteins inferred from 853 amino acid sequences using Maximum Likelihood method. The eight *M. circinelloides* Pdr proteins, *C. neoformand* CnAfr1 and the *C. albicans* Cdr1 and Cdr2 are indicated with bold characters. Numbers above or below branches are ultrafast bootstrap supports. Only values greater than 95% are shown.

A phylogeny was inferred from 853 amino acid sequences of PDR proteins representing the five main fungal phyla. A simplified view of the resulting tree is shown in **Figure 1** while the whole version can be found in **Supplementary Figure 3**. On this tree, PDR transporters of fungi belonging to the Chytridiomycota, Zoopagomycota and Mucoromycota (including the examined PDR1-8 of *M. circinelloides*) can be found in the clades, which clearly correspond to the phylogenetic position of these fungal groups. At the same time, Ascomycota and Basidiomycota sequences grouped together in various clades corresponding to different subfamilies of PDR proteins. Our results support the monophyly of Mucoromycota PDR proteins, which form a sister group of the Dicarya PDRs. Within the latter group the clade containing the *C. neoformans* Afr1 protein proved to be the closest to the Mucoromycota clade. Within the Mucoromycota clade, two main clades can be discriminated, among which the clade A contains the *M. circinelloides* PDR1, 6, 7 and 8 proteins while the clade B includes PDR2, 3, 4 and 5. Within the two main clades, proteins of a same species can be positioned in various subclades indicating several gene duplications and a diversification of the pdr genes in this fungal group.

### Relative Transcript Levels of the *pdr* Genes After Azole Treatment

**Figure 2:**
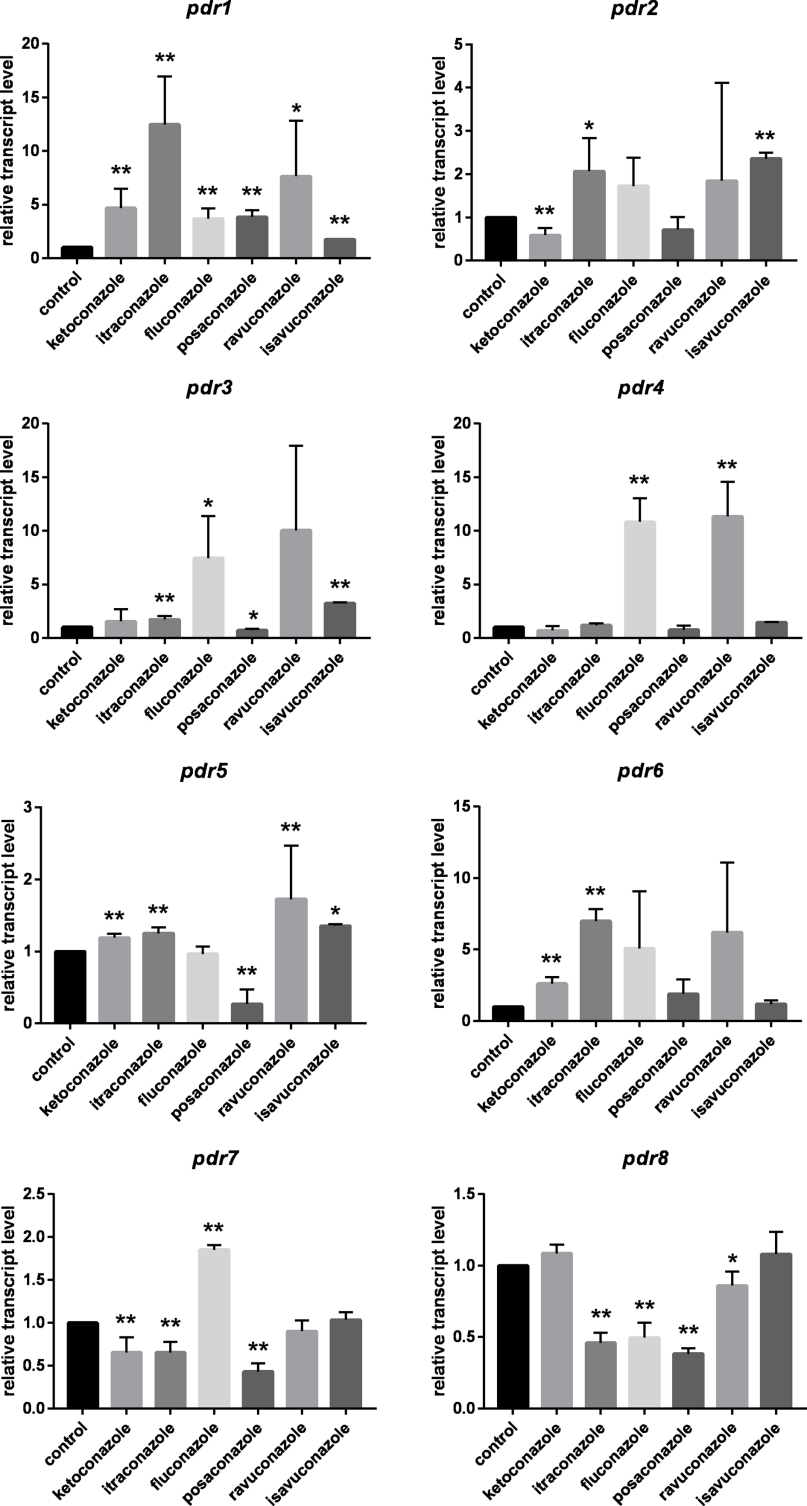
Relative transcript levels of the *pdr* genes of *M. circinelloides* after azole treatment. MS12 was grown in RPMI-1640 liquid media at 25°C; transcript level of each gene measured in the untreated control was taken as 1. The presented values are averages of three independent experiments; error bars indicate standard deviation. Relative transcript values followed by * and ** significantly differed from the untreated control according to the paired t-test (*p < 0.05 and **p < 0.01).

**Figure 3:**
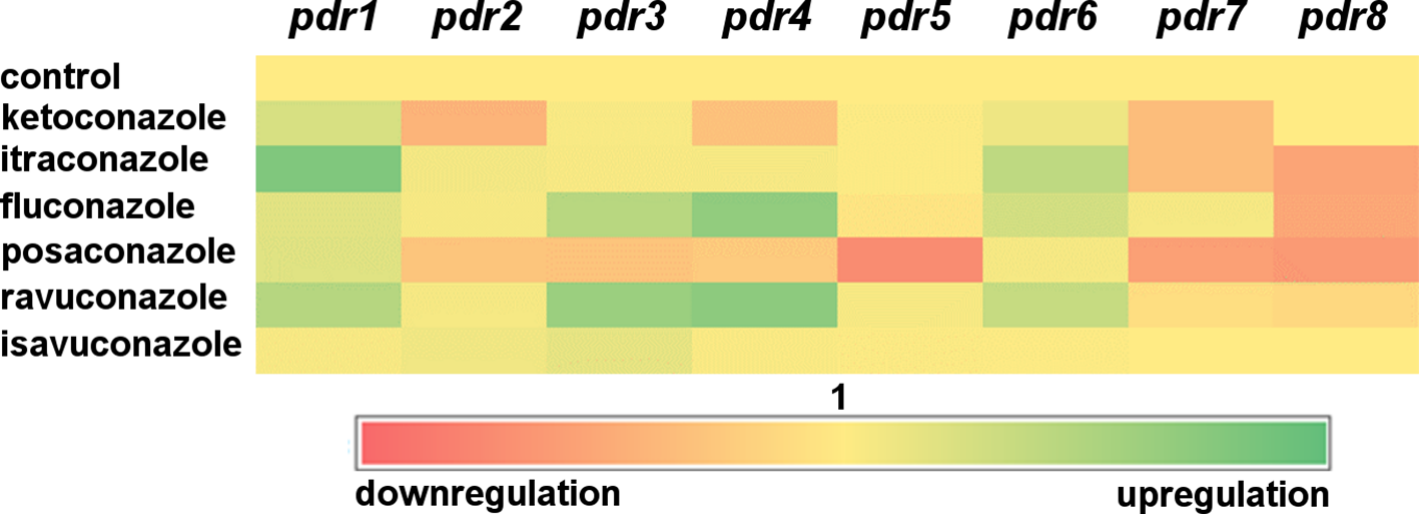
Heat-map of transcript level of *pdr* genes in response to exposure to azoles. Red and green colors indicate down- and upregulation, respectively, while yellow corresponds to the transcription activity of the control.

In general, *pdr* genes responded differently to the tested azoles (**Figure 2**). Itraconazole had the widest effect on *pdr* expression upregulating five of the eight genes, i.e., *pdr1*, *pdr2*, *pdr3*, *pdr5* and *pdr6*. Three genes (*pdr1*, *pdr5* and *pdr6*) displayed increased transcript levels in response to ketoconazole, while fluconazole treatment resulted in increased transcript level for *pdr1*, *pdr3*, *pdr4* and *pdr7*. Transcript level of *pdr1*, *pdr2*, *pdr3* and *pdr5* increased significantly after isavuconazole treatment. From the eight genes, only *pdr1* had significantly increased transcript levels in response to all azoles (**Figure 2**). Interestingly only this gene responded with higher transcript level to posaconazole. Treatments with ketoconazole, itraconazole, fluconazole, posaconazole and ravuconazole resulted in decreased transcript levels for *pdr7* and *pdr8*. *Pdr4* only responded to treatment with fluconazole and ravuconazole, while the *pdr6* transcript level significantly increased after ketoconazole and itraconazole treatments. A heat-map was generated to summarize the result of the qRT-PCR experiments (**Figure 3**). It can be seen that *pdr1*, *pdr3*, *pdr4* and *pdr6* are generally upregulated in response to azole treatments while *pdr2*, *pdr5* and especially *pdr7* and *pdr8* are rather downregulated by the azoles.

### Relative Transcript Levels of *pdr* Genes Under Anaerobic Condition

**Figure 4:**
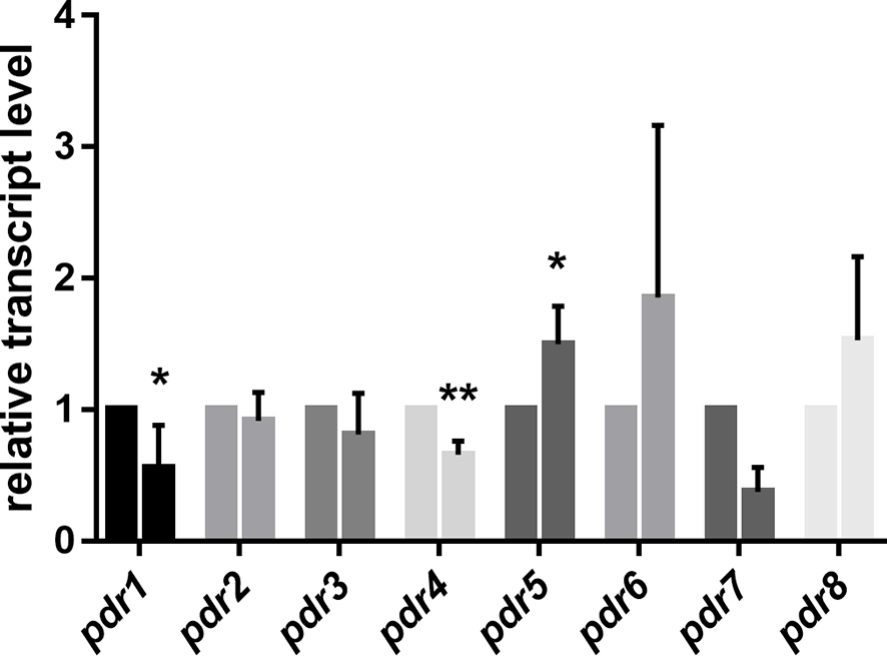
Relative transcript levels of *pdr* genes under anaerobic condition. Transcript level of each gene was measured under aerobic condition was taken as 1. The presented values are averages of three independent experiments; error bars indicate standard deviation. For RNA extraction, fungal strains were cultivated on YNB for two days at 25°C. Relative transcript values followed by * and ** significantly differed from the value taken as 1 according to the paired t-test (*p < 0.05 and **p < 0,01).

Morphological dimorphism is a characteristic feature of *M. circinelloides*. In the absence of oxygen, filamentous growth of the fungus switches to a yeast-like form. MS12 was grown in liquid RPMI-1640 at 25°C for 24 h. Then, the culture was transferred into an oxygen-free environment containing 10% CO_2_. RNA was extracted after incubating the fungus anaerobically for 240 min. *Pdr1* and *pdr3* displayed significantly decreased transcript levels under anaerobiosis compared to their transcript levels measured in aerobic environment (**Figure 4**). Contrarily, the transcript level of *pdr5* significantly increased under anaerobiosis (**Figure 4**).

### Knock Out of the *pdr1* Gene Using the CRISPR-Cas9 Method

Based on the qPCR experiment, deletion of *pdr1* gene was assigned for gene knock out by using the CRISPR-Cas9 technique. For deletion of *pdr1*, transformation and the genome editing frequencies were 4/10^5^ protoplasts and 100%, respectively, as demonstrated by PCR analysis of the isolated colonies amplifying the expected fragments (**Supplementary Table 3**, **Supplementary Figure 4**). Mutants proved to be mitotically stable retaining the integrated fragment even after 10 cultivation cycles. From the transformation experiment two isolates (MS12-Δ*pdr1*/1 and 2) were selected for the further experiments.

### Relative Transcript Levels of the Intact *pdr* Genes in the *Δpdr1* Knock Out Mutants

**Figure 5:**
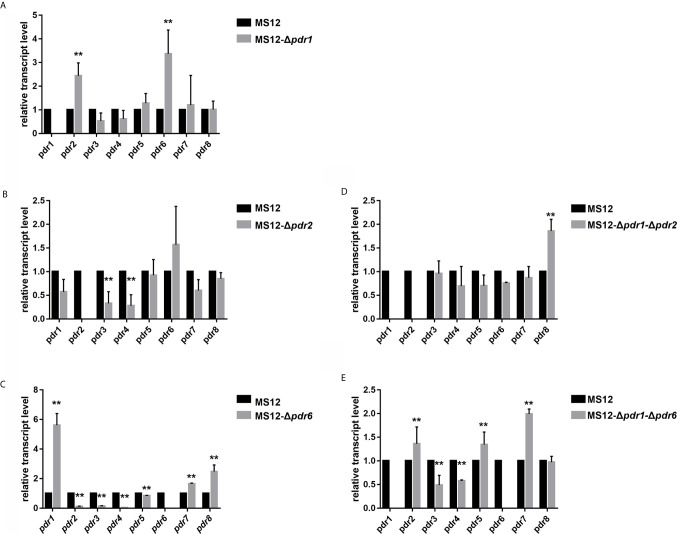
Relative transcript levels of *pdr* genes in the MS12-Δ*pdr1*/1 **(A)**, MS12-Δ*pdr2* **(B)**, MS12-Δ*pdr6* **(C)** MS12-Δ*pdr1*-Δ*pdr2* **(D)** and MS12-Δ*pdr1*-Δ*pdr6* **(E)** mutant compared to that in the original strain. Transcript level of each gene measured in the strain MS12 (black column) was taken as 1. The presented values are averages of three independent experiments (error bars indicate standard deviation). For RNA extraction, fungal strains were cultivated on YNB for two days at 25°C. Relative transcript levels significantly different from the value taken as 1 according to the paired *t*-test are indicated with * or ** (^*^ *p* < 0.05, ^**^ *p* < 0.01).

qRT-PCR analysis proved the absence of the transcript of the deleted *pdr1* gene and revealed that the relative transcript levels of *pdr2* and *pdr6* changed significantly in the mutant (**Figure 5A**).

### Deletion of *pdr2* and *pdr6* and Creation of Δ*pdr1*-Δ*pdr2* and Δ*pdr1*-Δ*pdr6* Double Knock out Mutants

Based on the qRT-PCR analysis of the MS12-Δ*pdr1* mutant strain, deletion of *pdr2* and *pdr6* gene was performed using the CRISPR-Cas9 system to create single and double mutants. For the MS12-Δ*pdr2* and MS12-Δ*pdr6* single mutants, transformation frequencies were 12 and 10 colonies per 10^5^ protoplasts, respectively while the genome editing efficiency was 83% and 100%, respectively (**Supplementary Table 3**, **Supplementary Figure 5**). To create Δ*pdr1*-Δ*pdr2* -and Δ*pdr1* Δ*pdr6* double mutants, MS12-Δ*pdr1* was used as the recipient strain. Transformation frequency was 8 and 3 colonies and the genome editing efficiency was 75% and 66.66% for MS12-Δ*pdr1*-Δ*pdr2* and MS12-Δ*pdr1*-Δ*pdr6*, respectively (**Supplementary Table 3**). Two isolates from each the transformation experiments were analyzed by PCR and the expected fragments were amplified (**Supplementary Figure 6**).

### Relative Transcript Levels of the Intact *pdr* Genes in the MS12-Δ*pdr2* and MS12-Δ*pdr6* Single-, and the MS12-Δ*pdr1*-Δ*pdr2* and MS12-Δ*pdr1*-Δ*pdr6* Double Knock Out Mutants

qRT-PCR analysis proved the absence of the transcripts of the deleted genes in all mutants. It also revealed that the transcript level of *pdr3* and *pdr4* significantly decreased in the MS12-Δ*pdr2* strain (**Figure 5B**). At the same time, deletion of the *pdr6* gene led to significantly increased relative transcript levels for *pdr1*, *pdr7* and *pdr8*, while the relative transcript level of the other *pdr* genes significantly decreased (**Figure 5C**).

After simultaneous deletion of *pdr1* and *pdr2*, the transcript level of *pdr8* significantly increased in the mutants (**Figure 5D**). In the MS12-Δ*pdr1*-Δ*pdr6* mutant, the transcript levels of *pdr2*, *pdr3*, *pdr5*, *pdr7* significantly increased, while those of *pdr4* and *pdr8* significantly decreased (**Figure 5E**).

### Effect of the *pdr1*, *pdr2*, and *pdr6* Gene Deletions on the Colony Growth of the Mutant Strains

**Figure 6:**
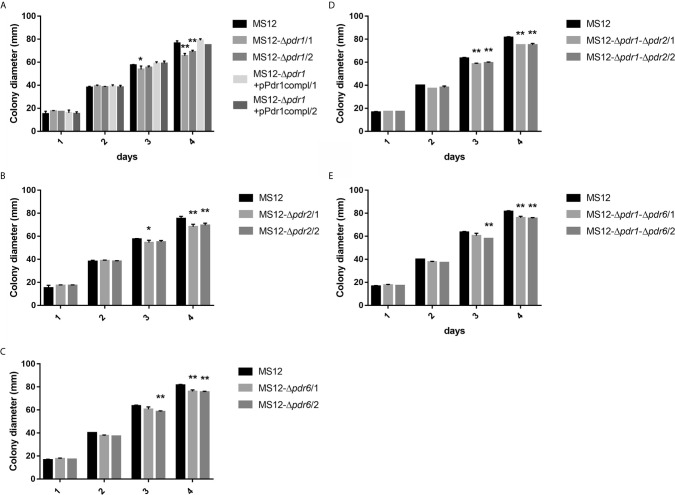
Colony diameters of the *pdr* deletion mutants and the original MS12 strain of *Mucor circinelloides* at 25°C on YNB medium. **(A)** shows the colony diameter of *pdr1* deleted and complemented strains, **(B, C)** show the colony diameter of the single *pdr2* and *pdr6* knock out mutants, while **(D, E)** show the colony diameter of the double knock out strains. The presented values are averages; colony diameters were measured during three independent cultivation (error bars indicate standard deviation). Values followed by * and ** significantly differed from the corresponding value of the MS12 strain according to the two-way Anova (*p < 0.05; **p < 0.01).

Colony growth of the single (i.e., MS12-Δ*pdr1*, MS12-Δ*pdr2* and MS12-Δ*pdr6*) and double *pdr* mutants (i.e., MS12-Δ*pdr1*-Δ*pdr2* and MS12-Δ*pdr1*-Δ*pdr6*) significantly decreased compared to the parental MS12 strain on the third and fourth days of cultivation (**Figure 6**). When deletion of the *pdr1* gene was complemented by transforming pPdr1compl plasmid into the MS12-Δ*pdr1* strain, growth ability of the resulting MS12-Δ*pdr1*+pPdr1compl strain proved to be similar to that of the original MS12 strain (**Figure 6A**).

### Measurement of the R6G Efflux Activity of the Tested Strains

**Figure 7:**
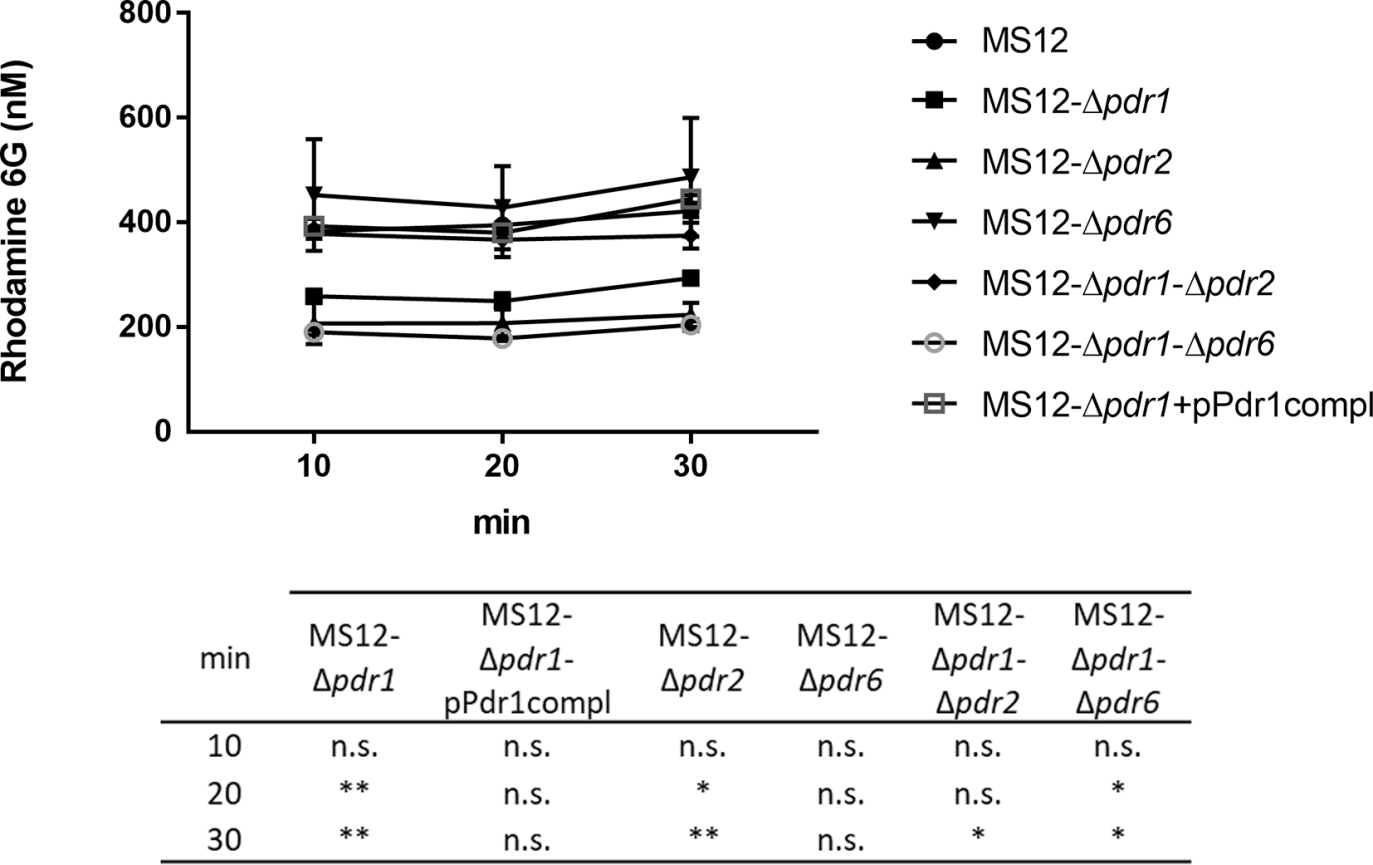
Measurement of the R6G efflux activity. After uptake of rhodamine 6G, fungal cells were resuspended in PBS supplemented with 2 nM of D-glucose. Values followed by * and ** significantly differed from the corresponding value of the MS12 strain according to the two-way Anova (*p < 0.05; **p < 0.01), n.s. is non-significant.

To examine the drug efflux activity of the strains, R6G efflux in the parental and the *pdr* mutant strains were compared. Twenty min after R6G uptake, MS12-Δ*pdr1* and MS12-Δ*pdr2* mutants showed significantly decreased efflux activity than the parental strain (**Figure 7**). After 30 min, efflux activity of all mutants, except MS12-Δ*pdr6*, significantly decreased (**Figure 7**).

### Effect of the Gene Knock Out on the Susceptibility to Different Antifungal Agents and Hydrogen Peroxide Under Aerobic and Anaerobic Conditions

Sensitivity of the MS12-Δ*pdr1*, MS12-Δ*pdr2*, MS12-Δ*pdr6*, MS12-Δ*pdr1*-Δ*pdr2* and MS12-Δ*pdr1*-Δ*pdr6* mutants and the MS12 strain were determined to different antifungal agents and hydrogen peroxide (H_2_O_2_) by using the broth microdilution method under aerobic and anaerobic conditions and the minimal inhibitory concentration (MIC) of different azoles was determined. Sensitivity of the mutants did not differ from that of the original MS12 strain to amphotericin B, hygromycin B, terbinafine, micafungin, ketoconazole, fluconazole, cycloheximide and H_2_O_2_ under both aerobic and anaerobic conditions. All mutants showed, however, increased susceptibility to posaconazole and ravuconazole, while knock out of *pdr1* resulted in increased sensitivity to isavuconazole under anaerobic condition as well (**Table 2**). After complementation of the *pdr1* deletion, susceptibility of MS12-Δ*pdr1*+pPdr1compl strains to all tested azoles was similar to that of the original MS12 strain.

**Table 2:** Minimal inhibitory concentrations (MIC) of the azoles (µg/ml) against the mutants and the parental *M. circinelloides* MS12 strain under aerobic condition.

MIC (µg/ml)						
Strain	Ketoconazole	Itraconazole	Fluconazole	Posaconazole	Ravuconazole	Isavuconazole
MS12	>64	>64	>64	4	16	>16
MS12-Δ*pdr1*/1	>64	>64	>64	2	4	16
MS12-Δ*pdr1*/2	>64	>64	>64	2	8	8
MS12-Δ*pdr1*+pPdr1compl/1	>64	>64	>64	4	>16	>16
MS12-Δ*pdr1*+pPdr1compl/2	>64	>64	>64	4	16	16
MS12-Δ*pdr2*/1	>64	>64	>64	2	16	>16
MS12-Δ*pdr2*/2	64	>64	>64	2	8	>16
MS12-Δ*pdr6*/1	>64	>64	>64	4	16 (MIC^50^)	16 (MIC^50^)
MS12-Δ*pdr6*/2	>64	>64	>64	4	16 (MIC^50^)	16 (MIC^50^)
MS12-Δ*pdr1-* Δ*pdr2*/1	>64	>64	>64	2	8	16
MS12-Δ*pdr1-* Δ*pdr2*/2	>64	>64	>64	2	16	16
MS12-Δ*pdr1-* Δ*pdr6*/1	>64	>64	>64	2	8	16
MS12-Δ*pdr1-* Δ*pdr6*/2	>64	>64	>64	2	8	16

Under anaerobic condition, knock out of *pdr2* resulted in decreased sensitivity to ketoconazole compared to the parental strain, while susceptibility of the MS12-Δ*pdr1* and MS12-Δ*pdr2* mutants increased to itraconazole, ravuconazole and isavuconazole. Deletion of *pdr1* and *pdr2* had no effect on the sensitivity to posaconazole under anaerobiosis. Knock out of *pdr6* had no effect on the susceptibility to the different azoles at all under anaerobiosis (**Table 3**).

**Table 3:** Minimal inhibitory concentrations (MIC) of the azoles (µg/ml) against the transformants and the parental *M. circinelloides* MS12 strain under anaerobic condition.

MIC (µg/ml)						
Strain	Ketoconazole	Itraconazole	Fluconazole	Posaconazole	Ravuconazole	Isavuconazole
MS12	32	4	>64	2	4	4
MS12-Δ*pdr1*/1	32	2	>64	2	2	2
MS12-Δ*pdr1*/2	32	2	>64	2	2	2
MS12-Δ*pdr1*+pPdr1compl/1	32	4	>64	2	4	4
MS12-Δ*pdr1*+pPdr1compl/1	32	4	>64	2	2	2
MS12-Δ*pdr2*/1	64	2	>64	2	2	4
MS12-Δ*pdr2*/2	64	2	>64	1	2	2
MS12-Δ*pdr6*/1	32	4	>64	2	4	4
MS12-Δ*pdr6*/2	32	4	>64	2	4	4
MS12-Δ*pdr1-* Δ*pdr2*/1	32	2	>64	2	2	2
MS12-Δ*pdr1-* Δ*pdr2*/2	32	2	>64	2	2	2
MS12-Δ*pdr1-* Δ*pdr6*/1	64	2	>64	2	2	4
MS12-Δ*pdr1-* Δ*pdr6*/2	64	2	>64	1	2	2

## Discussion

Treatment of mucormycosis is looked upon as challenging and aggravated by the fact that Mucorales species are resistant to most azole antifungals. The molecular mechanism of their innate resistance to short-tailed azoles was described, but yet not completely clarified (Caramalho et al., 2017). Resistance mechanisms of various *Candida* and *Aspergillus* species have been characterized with more details (Cuenca-Estrella, 2014). The main target of azoles is the lanosterol-demethylase enzyme (i.e. Cyp51 or Erg11), which plays a crucial role in the ergosterol biosynthesis of yeasts and molds (Monk et al., 2020). Overexpression of and point mutations in the *cyp51* gene can cause increased resistance to different azoles used in the clinic and agriculture (Parker et al., 2014). Besides lanosterol-demethylase, ABC- and MFS transporters are involved in the mechanism of azole resistance (Fraczek et al., 2013; Cuenca-Estrella, 2014; Gonçalves et al., 2016; Rocha et al., 2017). In *C. albicans*, the MFS-transporter MDR1 contribute to the fluconazole, voriconazole and ketoconazole resistance (Morschhäuser, 2010), but does not confer resistance to posaconazole, isavuconazole or itraconazole (Sanglard and Coste, 2015).

ABC transporters constitute one of the largest transporter superfamily. These proteins often consist of multiple subunits (i.e., of the transmembrane domain [TMD] and the nucleotide binding domain [NBD]). The transport is driven by ATP hydrolysis (Wilkens, 2015). ATP is the substrate of NBD, which has some conserved motifs, such as Walker-A, Walker-B and the ABC-signature (Seeger and van Veen, 2009; Lamping et al., 2010; Holmes et al., 2016). The sequence of TMD is more diverse reflecting to the diversity of the transported substrate (Seeger and van Veen, 2009). Within the ABC transporters, the PDR subfamily is the most closely associated to drug resistance (Cannon et al., 2009). In the *M. circinelloides* genome database, eight putative PDR transporter coding genes were identified. The abovementioned motifs characteristic to ABC transporters were identified in each of them.

Analysis of the promoter regions of the eight *Mucor* genes did not reveal the presence of the motifs described as PDRE (TCCG/aC/tGG/cA) or CDRE (CGGA(A/T)ATCGGATATTTTTTTT) elements. These motifs were originally found and characterized in *Saccharomyces* (Mamnun et al., 2002) and *Candida* (de Micheli et al., 2002) as the binding sites of transcription factors regulating the expression of the *pdr* genes. If such binding sites are present, the consensus sequences established for Ascomycota cannot be applied to find them, maybe because of the large evolutionary distance.

Phylogenetic analysis of PDR transporters indicated the monophyly of Mucoromycota PDRs; although, within the Mucoromycota clade various gene duplication events can be assumed. Based on a phylogenetic analysis of 78 PDR proteins, Lamping et al. (2010) previously discerned 10 clades of PDR transporters within the Dicarya, among which four clades (i.e. clades B, G, H1a and H1b) contained PDRs of both Ascomycota and Basidiomycota fungi. None of the involved Mucoromycota sequences grouped together with any of the Dikarya PDRs.

*Mucor pdr* genes show similarity to the *C. neoformans Afr1* gene, which was also strengthen by the phylogenetic analysis of the fungal PDR genes *Afr1* is involved in the resistance of *Cryptococcus* to fluconazole (Sanguinetti et al., 2006). Overexpression of *Afr1* increased the azole resistance indicating that upregulation of the gene may play a role in the fluconazole resistance (Posteraro et al., 2003; Sanguinetti et al., 2006; Morschhäuser, 2010). In our experiments, transcription level of all *M. circinelloides pdr* genes altered after treatment with either of the tested azoles, although only that of the *pdr1* increased in response to all of them.

Mucorales species are generally resistant to fluconazole and voriconazole, while isavuconazole and itraconazole were found to have *in vitro* species-specific activity (Dannaoui, 2017). International guidelines for the treatment of mucormycosis suggest first-line antifungal therapy with lipid formulations of amphotericin B (AMB), whereas posaconazole (Cornely et al., 2014) is recommended and isavuconazole (Ashkenazi-Hoffnung et al., 2020) was successfully used as salvage therapy. In our study, posaconazole treatment affected the transcription of *pdr1* only while isavuconazole significantly increased the transcript level of *pdr1*, *pdr2*, *pdr3* and *pdr5*.

Deletion of *pdr1* increased the sensitivity of the mutants to posaconazole and isavuconazole. Although it was found to be upregulated by isavuconazole, deletion of *pdr2* did not affect the susceptibility to this compound. At the same time, MS12-Δ*pdr2* mutants showed increased susceptibility to posaconazole similarly to the MS12-Δ*pdr1* strains.

Fluconazole treatment affected the transcription of various genes including *pdr1* but neither single nor double deletion mutants generated in this study displayed altered susceptibility to this drug. This result is in agreement with the observations that, in addition to active transport mechanisms, alterations in the lanosterol-demethylase gene (Edlind et al., 2001; Sionov et al., 2012; Leonardelli et al., 2016; Caramalho et al., 2017) and/or a novel Zn2-Cys6 transcription factor may also underlie the fluconazole resistance of these fungi (Hagiwara et al., 2017).

qRT-PCR analysis revealed highly similar transcription patterns for *pdr1* and *pdr6* after azole treatment. At the same time, deletion of *pdr1* caused significantly increased transcript level of *pdr2* and *pdr6*, while the relative transcript level of *pdr1*, *pdr7* and *pdr8* significantly increased in the MS12-Δ*pdr6* mutant. These results suggest that regulation of *pdr* genes is highly interconnected and coordinated. It can also be assumed that the increased activity of certain *pdr* genes may compensate the lack of the deleted genes. A similar phenomenon was previously described for the three HMG-CoA reductase isogenes in *M. circinelloides* (Nagy et al., 2019). Functional linkages of genes involved in antifungal resistance was observed in *Saccharomyces cerevisiae* where deletion of two multidrug transporter genes, *yor1* and *snq2* caused an increased expression of *pdr5*, while the lack of *pdr5* and *snq2* induced *yor1* expression (Kolaczkowska et al., 2008).

Susceptibility testing of the deletion mutants suggests that *pdr1* and *pdr2* may have role in the protection from the effect of posaconazole, isavuconazole and ravuconazole and this function is affected by the oxygen level of the environment. At the same time, deletion of *pdr6* had no significant effect on the azole resistance of the fungus. Deletion of *pdr1* and *pdr2* resulted decreased efflux activity in all tested mutant strains, while the efflux activity of MS12-Δ*pdr6* strain was similar to the parental MS12 strain.

Our results suggest that the tested *pdr* genes involved in different degrees in the resistance to the different azoles. In *C. glabrata* Cdr1 and Cdr2 also had a different effect on the resistance to azoles (Sanglard et al., 2001). In *A. fumigatus* deletion of *abcA* and *abcB/cdr1B* transporters, which show the highest similarity to *S. cerevisiae pdr5*, resulted in increased susceptibility to voriconazole, but this effect was more pronounced in case of the Δ*abcB/cdr1B* mutant (Paul et al., 2013). In *Aspergillus oryzae*, deletion of *atrG* belonging to the PDR subfamily, caused increased azole susceptibility, while its simultaneous deletion with *atrA* conferred azole hypersensitivity (Miura et al., 2018).

In the absence of oxygen, filamentous growth of *M. circinelloides* switches to a yeast-like form and the fungus produces energy by fermentation (Nagy et al., 2014). These morphological and metabolic changes are necessary associated to serious changes in the expression of numerous genes. Transporters and other membrane associated proteins are expected to be affected by these processes. Under anaerobic condition, the transcript levels of *pdr1* and *pdr4* decreased, while the transcript level of *pdr5* increased significantly compared to the aerobic condition. The transcript levels of other *pdr* genes did not change significantly. The growth ability of the created mutants somewhat decreased compared to the parental strain. This phenomenon can be explained by the fact that the function of PDR transporters in not limited to the drug export. In *S. cerevisiae* Aus1p and Pdr11p are required for import of exogenous sterols for anaerobic growth (Wilcox et al., 2002; Reiner et al., 2006; Kohut et al., 2011; Zavrel et al., 2013) and simultaneous disruption of the encoding genes proved to be lethal under anaerobiosis (Wilcox et al., 2002). *S. cerevisiae* Pdr5 and Yor1 play role in the externalization of lipids (Decottignies et al., 1998; Pomorski et al., 2003). In *C. albicans*, Cdr1 plays role in the drug efflux and, along with Cdr2 and Cdr3, involved in the externalization of lipids (Smriti et al., 2002; Tsao et al., 2009).

## Conclusion

*M. circinelloides* has eight genes encoding PDR transporters. Transcription analysis of these genes suggests that their regulation is interconnected and affected by the different azoles. The genes *pdr1* and *pdr2* seem to participate in the resistance or reduced sensitivity to posaconazole, ravuconazole and isavuconazole. It is also clear, that resistance to these azoles and azole resistance in general cannot be completely explained by the activity of the tested PDR proteins. Other mechanisms, such as special regulatory processes, multiple transporter genes and alterations in the lanosterol-demethylase gene might also be involved.

## Data Availability Statement

The original contributions presented in the study are included in the article/**Supplementary Material**. Further inquiries can be directed to the corresponding author.

## Author Contributions

GN (1st Author) and TP designed the approach and the experiments, participated in the experimental work, managed the study, and wrote the manuscript. SKi, RV, KB, CS, HM, ZN, and GN (10th Author) participated in the experimental work. SKo, LB, and CV participated in the data analysis, evaluation of the results and drafting the manuscript. All authors contributed to the article and approved the submitted version.

## Funding

This study was supported by the “Lendület” Grant of the Hungarian Academy of Sciences (LP2016-8/2016), the project GINOP-2.3.2-15-2016-00035 and the NKFI project K131796. GN is grateful for support of the Premium Postdoctoral Fellowship Program of the Hungarian Academy of Sciences (460050). LB was supported by the János Bolyai Research Scholarship (BO/00522/19/8) of the Hungarian Academy of Sciences.

## Conflict of Interest

The authors declare that the research was conducted in the absence of any commercial or financial relationships that could be construed as a potential conflict of interest.
