# Supplementary material for: Characterization of Three Pleiotropic Drug Resistance Transporter Genes and Their Participation in the Azole Resistance of Mucor circinelloides
Source: Front Cell Infect Microbiol. 2021 Apr 14;11:660347. doi: 10.3389/fcimb.2021.660347 (PMC8079984; doi:10.3389/fcimb.2021.660347)
Supplement: Supplementary file 1 [file DataSheet_1.pdf]

frontiers

Supplementary Material

Characterization of three pleiotropic drug resistance transporter genes and their participation in the azole resistance of *Mucor circinelloides*

Gábor Nagy[1,2], Sándor Kiss[1], Rakesh Varghese[1], Kitti Bauer[1], Csilla Szebenyi[1,2], Sándor Kocsubé[1], László Bodai[3], Nóra Zsindely[3], Gábor Nagy[3], Csaba Vágvölgyi[1], Tamás Papp[1,2]

Supplementary Table 1 Primers used in the present study.

Primers	Sequence 5’-3’	Amplified DNA
	**Phusion PCR for *pdr1* deletion**	
Mc48059p1	ACGGTGTCTTTCTTCTTATACTCTG	Promoter of *pdr1*
Mc48059p2	GATATTGACAGCATTGGCAGTGG	
Mc48059p3	CATTTGGTGAACCCACTGCCAATGCTGTCAATATCTGCCTCAGCATTGGTACTTG	*pyrG* with own promoter and terminator
Mc48059p4	TTAACGTCTAGGCTTTCTAAAGACATAAACAAGGCGTACACTGGCCATGCTATCG	
Mc48059p5	GCCTTGTTTATGTCTTTAGAAAGCC	Terminator of *pdr1*
Mc48059p6	AGACTACGTGACATTCTTGCTACC	
Mc48059p7	CTCTCCTAGCTCAAAGTAAGCA	Final PCR product for transformation
Mc48059p8	TCCAAGGCATTGTAAGTATCTG	
	**Phusion PCR for *pdr2* deletion**	
Mc83305/1	TAGTTTCACCTTTAAGCCATCC	Promoter of *pdr1*
Mc83305/2	GCCCTTATACCTTCAATCGCT	
Mc83305/3	CACTGTCTTTAATGTCATGCTC	Terminator of *pdr1*
Mc83305/4	TCCTGTAAATCCACAACAACTG	
Mc83305/5	ATGGATATGCCATCAGCGATTGAAGGTATAAGGGCCTGCAGGATGGGACAAGGTATATAT	*leuA* with own promoter and terminator
Mc83305/6	CAAAGAACATAAAGAGCATGACATTAAAGACAGTGCTGCAGTAGCTGTTGATGTTGTTGT	
Mc83305/7	GCATTCGCCAATAAAGTTCTG	Final PCR product for transformation
Mc83305/8	CTTCTACTAATGACCTAAGCGT	
	**Phusion PCR for *pdr6* deletion**	
Mc146716/1	CATTGACTGATAAACCTTGACCC	Promoter of *pdr6*
Mc146716/2	TCGCCATAAGTGCCATTTCTG	
Mc146716/3	CACTATCTGCTGCTAATTTCTTGTC	Terminator of *pdr6*
Mc146716/4	GCCCTTATACCTTCAATCGCT	
Mc146716/5	CAACTTTGGCAGAAATGGCACTTATGGCGACTGCAGGATGGGACAAGGTATATAT	

**Primers**	**Sequence 5’-3’**	**Amplified DNA**
Mc146716/6	CTTTGACAAGAAATTAGCAGCAGATAGTGCTGCAGTAGCTG TTGATGTTGTTGT	*leuA* with own promoter and terminator
Mc146716/7<br>Mc146716/8	GCATTCGCCAATAAAGTTCTG<br>CTTCTACTAATGACCTAAGCGT	Final PCR product for transformation
	**Primers for qRT-PCR experiments**	
Mc48059rtfw<br>Mc48059rtrev	ATCAACCCATTGGTCATTTCC<br>CAAATCGCCTTCATTACACTG	180 bp of *pdr1*
Mc83305fw<br>Mc83305rev	GTCAACCTCAAGAAGTGTCCA<br>GATACCGATACCAGCCTCCA	122 bp of *pdr2*
Mc141912rtfw<br>Mc141912rtrev	GCTCTGTACCATTCATCAACCC<br>GTCTTTGCCAATCTCACCGA	109 bp of *pdr3*
Mc142239rtfw<br>Mc142239rtrev	AAGCCTATGCACAATCCTTCTG<br>GTAAAGCCAGTAATCAAGCCGA	136 bp of *pdr4*
Mc145852rtfw<br>Mc145852rtrev	CTTACACCAACACTGACTACCA<br>GAAGATCGACATACTCCATAGCA	198 bp of *pdr5*
Mc146716rtfw<br>Mc146716rtrev	CATTTGCTGAACGACATCTCTG<br>AGACATTGCCTTCCACTTTACC	139 bp of *pdr6*
Mc158611rtfw<br>Mc158611rtrev	CTAATCCTGATGCTGTTCAACCT<br>TATCTTCTGCCCTTTCTCTTCCA	193 bp of *pdr7*
Mc186086rtfw<br>Mc186086rtrev	TCCTCCTTCAACATTATTCGCT<br>ATCCTGACCAATTTCACCGT	165 bp of *pdr8*
MCactinF<br>MCactinR	CACTCCTTCACTACCACCGCTGA<br>GAGAGCAGAGGATTGAGCAGCAG	117 bp of actin
	**Primers for complementation of *pdr1* deletion**	
pdr1fw<br>pdr1rev	TTAGCGGCCGCCTCTCCTAGCTCAAAGTAAGCA<br>TTAGCGGCCGCTCCAAGGCATTGTAAGTATCTG	*pdr1*gene with own promoter and terminator
leuAfw<br>leuArev	CTGCAGGATGGGACAAGGTATATAT<br>CTGCAGTAGCTGTTGATGTTGTTGT	leuA gene with own promoter and terminator

Underlined nucleotides indicate the recognition sequence of *Not*I.

Supplementary Table 2 Amino acid sequences of the 853 PDR proteins involved in the phylogenetic analysis.

Supplementray Table 3 Characteristics and efficiency of the CRISPR-Cas9 mediated genome editing in the creation of *pdr* mutant strains.

target	selection marker	transformation frequency (colonies/10^5 protoplast)	genome editing efficiency
pdr1	*pyrG*	4	100%
pdr2	*leuA*	12	83.33%
pdr6	*leuA*	10	100%
pdr1-pdr2	*pyrG* and *leuA*	8	75%
pdr1-pdr6	*pyrG* and *leuA*	3	66.66%

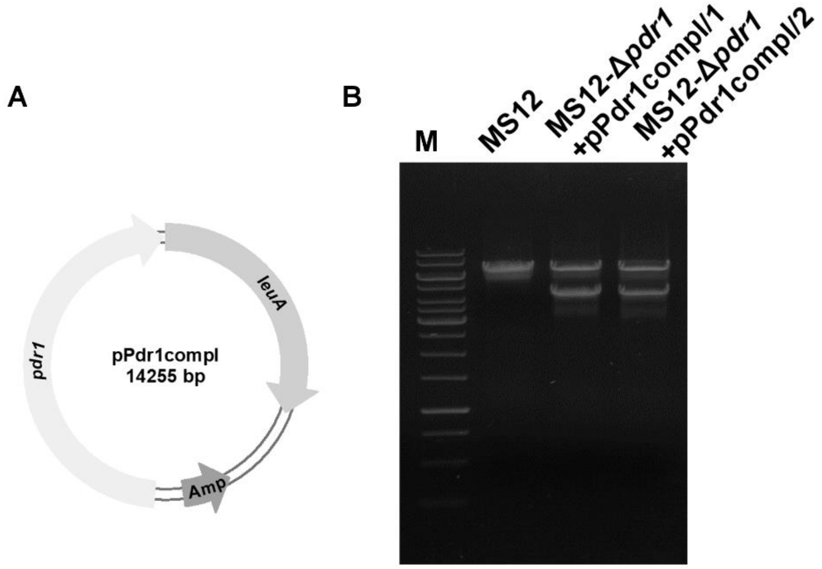

Supplementary Figure 1 Schematic picture of pPdr1compl vector using complementation of pdr1 deletion (A). Amplification of the *pdr1* gene with Mc48059P7 and Mc48059P8 primers (**Supplementary Table 1**) form the MS12-Δpdr1+pPdr1compl transformants (B). Colony diameter of the MS12-Δpdr1+pPdr1compl strain compared to the parental MS12 strain (C). Minimal inhibitory concentrations (MIC) of the azoles (μg/ml) against the MS12-Δpdr1+pPdr1compl strain and the parental *M. circinelloides* MS12 strain.

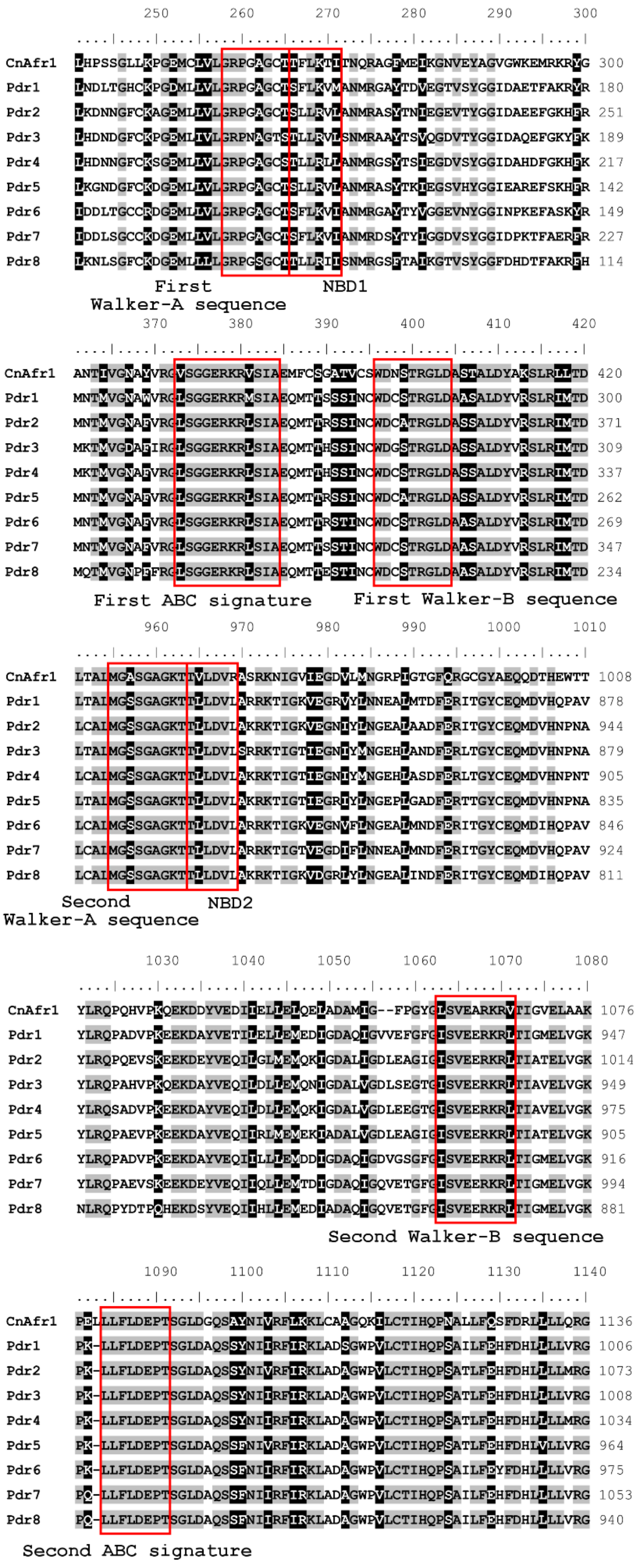
CnAfr1
Pdr1
Pdr2
Pdr3
Pdr4
Pdr5
Pdr6
Pdr7
Pdr8
First Walker-A sequence
NBD1
First ABC signature
First Walker-B sequence
Second Walker-A sequence
NBD2
Second Walker-B sequence
Second ABC signature

Supplementary Figure 2 Amino acid sequence alignment of the eight putative PDR protein of *Mucor circinelloides* and *C. neoformans* Afr1 protein. Grey background shows the identical and the black background show the similar amino acids. The red frame indicates the conserved motifs such as Walker-A, Walker-B and ABC signatures.

Supplementary Figure 3 Phylogeny of PDR proteins inferred from 853 amino acid sequences using the Maximum Likelihood method. Phylogenetic reconstruction was carried out by using IQ-TREE v. 1.6.12 with the LG4M+R6 model determined by the inbuilt model selection tool; branch supports were calculated with 5000 ultrafast bootstrap.

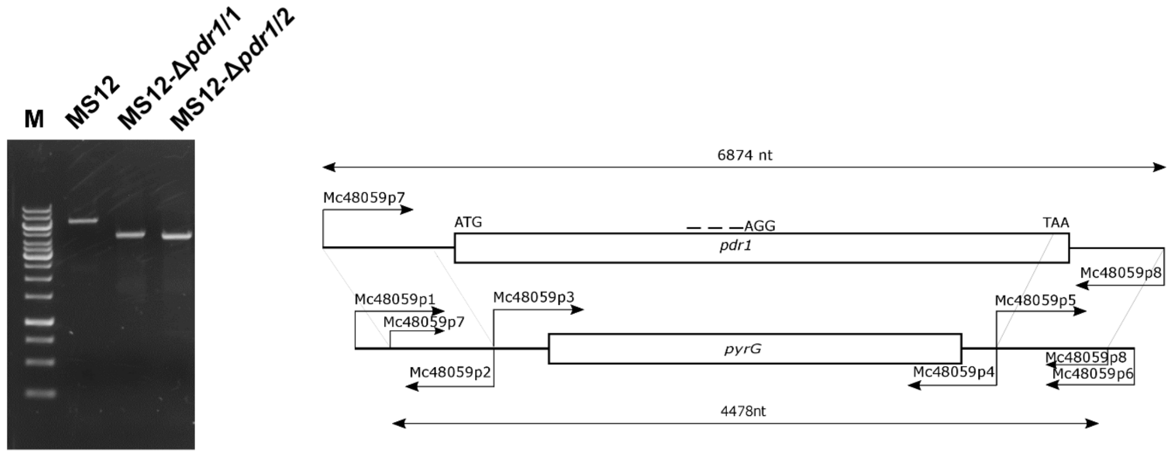

Supplementary Figure 4 PCR analysis of the transformants and the genome editing strategy designed to delete *pdr1* gene. For gele pictures M: GeneRuler 1 kb DNA ruler (Thermo Scientific), MS12 was the parental strain. In the PCR experiments, the primer pairs Mc48059p7-Mc48059p8 were used for *pdr1*. For genome editing strategies, HDR was performed using the disruption cassette as template DNA containing the *pyrG* gene as selection marker. Positions of the primers used to analyze or amplify the constructs are presented (for the nucleic acid sequences of the primers, see **Supplementary Table 1**). AGG indicates the PAM sequence while the arrows show the orientations of the primers.

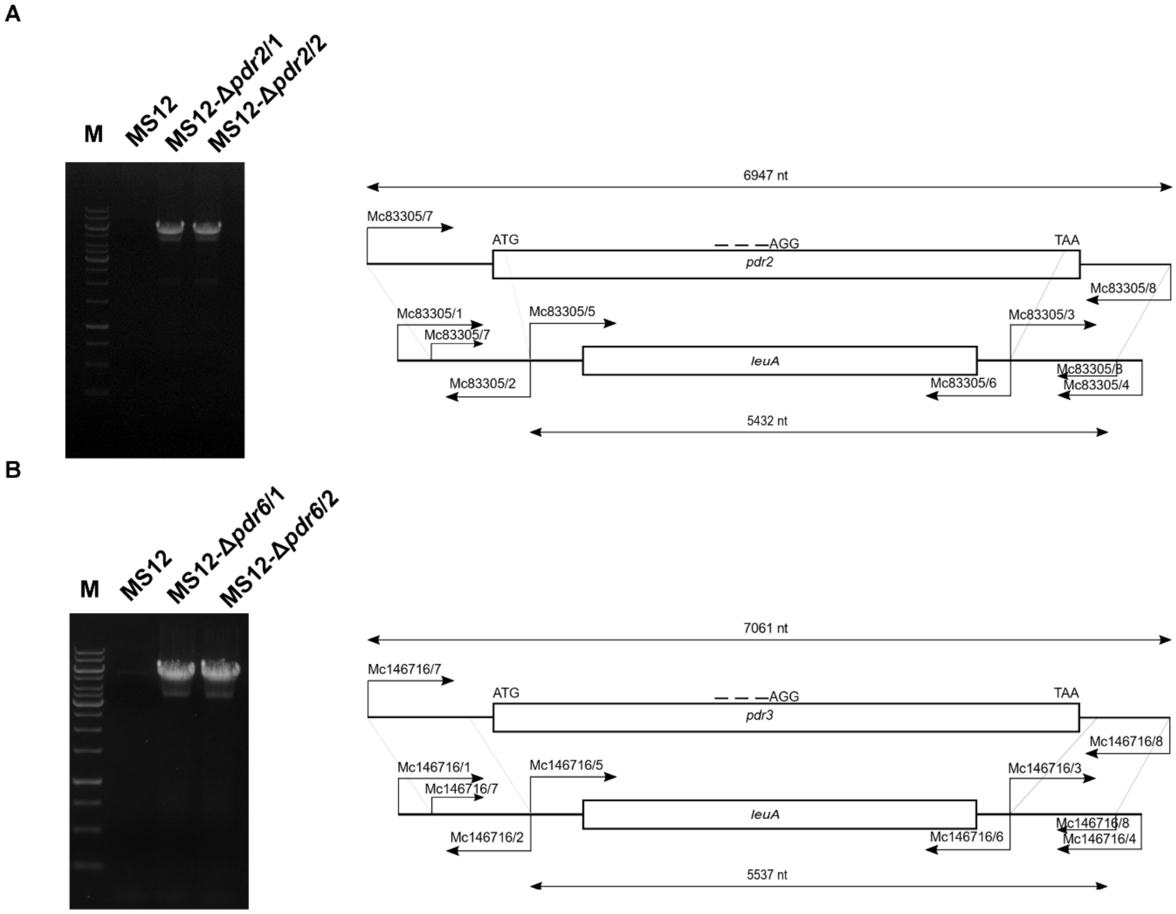

Supplementary Figure 5 PCR analysis of the transformants and the genome editing strategy designed to delete *pdr2* (A) and *pdr6* (B) genes. For gele pictures M: GeneRuler 1 kb DNA ruler (Thermo Scientific), MS12 was the parental strain. In the PCR experiments, the primer pairs Mc83305p5-Mc83305p8 for *pdr2* and Mc146716p5-Mc146716p8 were used for *pdr6*. For genome editing strategies, HDR was performed using the disruption cassette as template DNA containing the *leuA* gene as selection marker. Positions of the primers used to analyze or amplify the constructs are presented (for the nucleic acid sequences of the primers, see **Supplementary Table 1**). AGG indicates the PAM sequence while the arrows show the orientations of the primers.

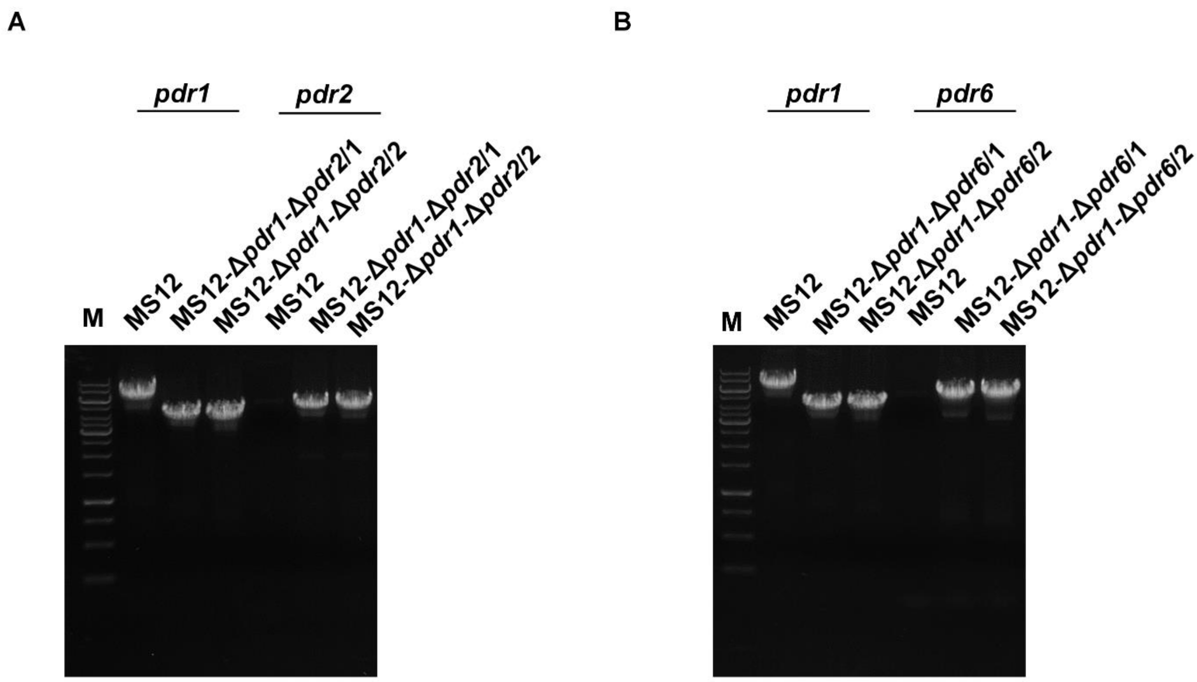

Supplementary Figure 6 PCR analysis of the transformants. For gele pictures M: GeneRuler 1 kb DNA ruler (Thermo Scientific), MS12 was the parental strain. In the PCR experiments, the primer pairs Mc48059p7-Mc48059p8 and Mc83305p5-Mc83305p8 for Δ*pdr1*-Δ*pdr2* (A) and Mc48059p7-Mc48059p8 and Mc146716p5-Mc146716p8 used for Δ*pdr1*-Δ*pdr6* (B).
