# Supplementary figures and images for: Characterization of Three Pleiotropic Drug Resistance Transporter Genes and Their Participation in the Azole Resistance of Mucor circinelloides
Source: Front Cell Infect Microbiol. 2021 Apr 14;11:660347. doi: 10.3389/fcimb.2021.660347 (PMC8079984; doi:10.3389/fcimb.2021.660347)

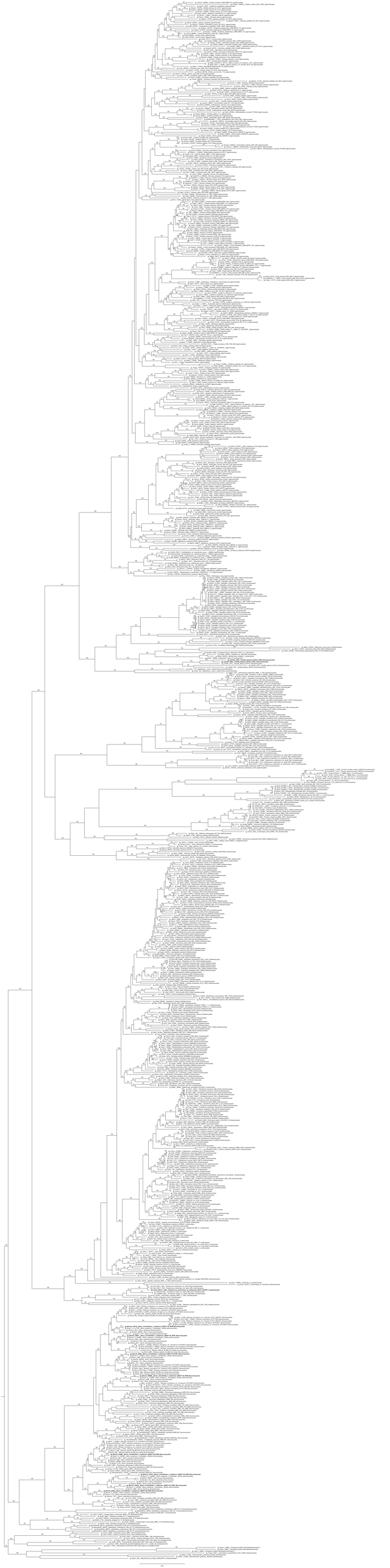

Supplement: Supplementary file 2 [file Image_2.pdf]
